# Supplementary material for: Predicting visual function by interpreting a neuronal wiring diagram
Source: Nature. 2024 Oct 2;634(8032):113–23. doi: 10.1038/s41586-024-07953-5 (PMC11446822; doi:10.1038/s41586-024-07953-5)
Supplement: Supplementary file 6 — CRF and ERF predictions for individual TmY4 and TmY9 cells. Analogous to Supplementary Data 3, but for TmY target types. Shown are the top four monosynaptic pathways, the strongest pathway passing through each of the top ten intermediary types (ranking from Extended Data Fig. 7), and the trisynaptic pathway Tm1–TmY–Dm3–TmY (see the section entitled Prediction of spatial normalization). [file 41586_2024_7953_MOESM6_ESM.zip › DataS4/TmY9qΓèÑ/720575940608376073.pdf]

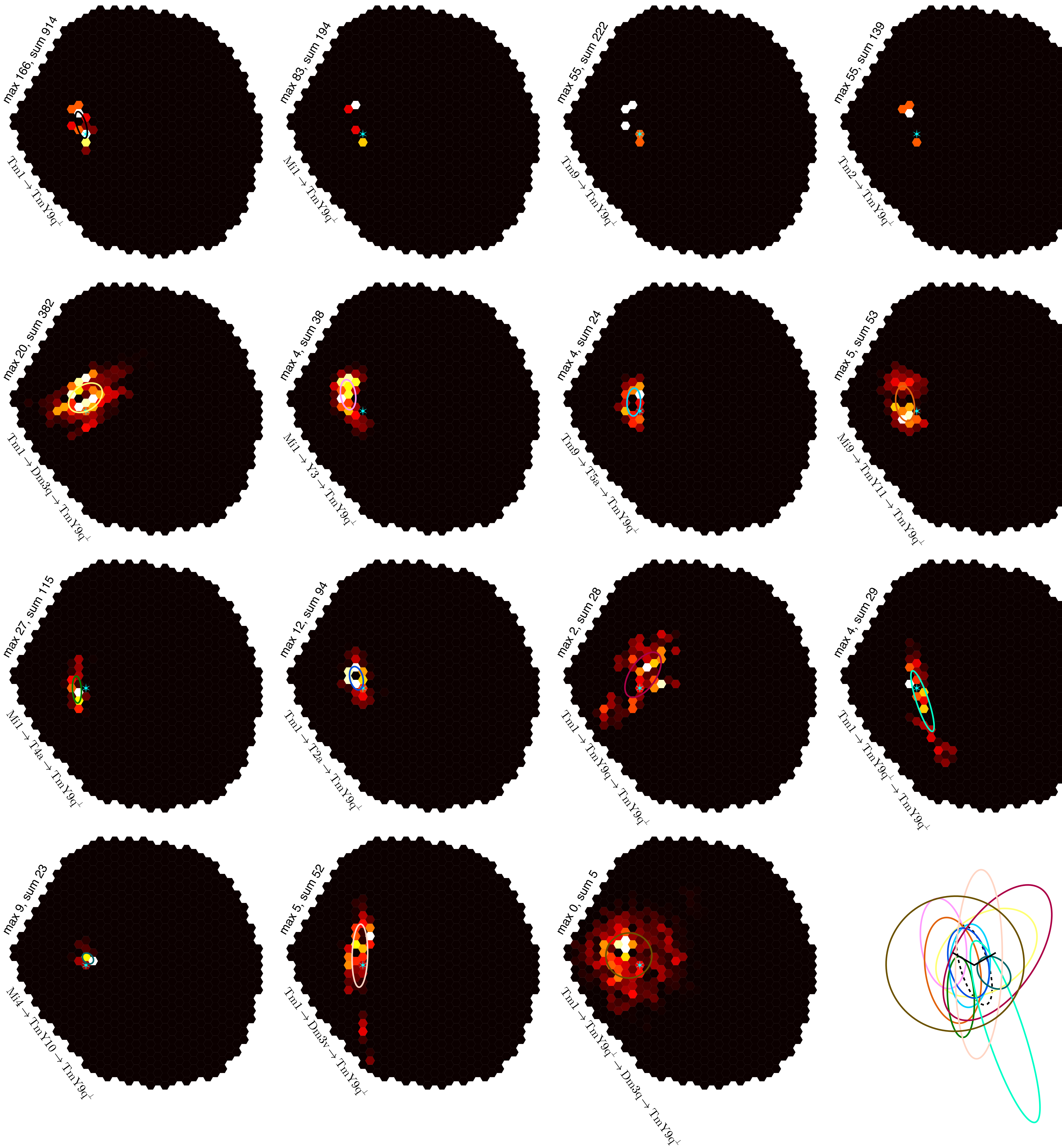
max 166, sum 914
Tm1 → TmY9q⊥
max 83, sum 194
Mi1 → TmY9q⊥
max 55, sum 222
Tm9 → TmY9q⊥
max 55, sum 139
Tm2 → TmY9q⊥
max 20, sum 382
Tm1 → Dm3q → TmY9q⊥
max 4, sum 38
Mi1 → Y3 → TmY9q⊥
max 4, sum 24
Tm9 → T5a → TmY9q⊥
max 5, sum 53
Mi9 → TmY11 → TmY9q⊥
max 27, sum 115
Mi1 → T4a → TmY9q⊥
max 12, sum 94
Tm1 → T2a → TmY9q⊥
max 2, sum 28
Tm1 → TmY9q → TmY9q⊥
max 4, sum 29
Tm1 → TmY9q⊥ → TmY9q⊥
max 9, sum 23
Mi4 → TmY10 → TmY9q⊥
max 5, sum 52
Tm1 → Dm3v → TmY9q⊥
max 0, sum 5
Tm1 → TmY9q⊥ → Dm3q → TmY9q⊥
